# Development of a Novel Medical Device for Mucositis and Peri-Implantitis Treatment

**DOI:** 10.3390/bioengineering7030087

**Published:** 2020-08-05

**Authors:** Gloria Cosoli, Lorenzo Scalise, Alfredo De Leo, Paola Russo, Gerardo Tricarico, Enrico Primo Tomasini, Graziano Cerri

**Affiliations:** 1 Department of Industrial Engineering and Mathematical Sciences, Università Politecnica delle Marche, 60131 Ancona, Italy; l.scalise@staff.univpm.it (L.S.); e.p.tomasini@staff.univpm.it (E.P.T.); 2 Department of Information Engineering, Università Politecnica delle Marche, 60131 Ancona, Italy; a.deleo@staff.univpm.it (A.D.L.); p.russo@staff.univpm.it (P.R.); g.cerri@staff.univpm.it (G.C.); 3 Department of Health Sciences, Università del Piemonte Orientale, 28100 Novara, Italy; gerardo.tricarico@aslvc.piemonte.it

**Keywords:** dentistry, dental implants, bioimpedance, electromagnetic compatibility, immunity testing, radio frequency, peri-implantitis, mucositis, peri-implant diseases, medical device

## Abstract

In spite of all the developments in dental implantology techniques, peri-implant diseases are frequent (prevalence up to 80% and 56% of subjects for mucositis and peri-implantitis, respectively) and there is an urgency for an effective treatment strategy. This paper presents an innovative electromedical device for the electromagnetic treatment of mucositis and peri-implantitis diseases. This device is also equipped with a measurement part for bioimpedance, which reflects the health conditions of a tissue, thus allowing clinicians to objectively detect impaired areas and to monitor the severity of the disease, evaluate the treatment efficacy, and adjust it accordingly. The design of the device was realized considering literature data, clinical evidence, numerical simulation results, and electromagnetic compatibility (EMC) pre-compliance tests, involving both clinicians and engineers, to better understand all the needs and translate them into design requirements. The reported system is being tested in more than 50 dental offices since 2019, providing efficient treatments for mucositis and peri-implantitis, with success rates of approximately 98% and 80%, respectively.

## 1. Introduction

Tooth loss is a very common problem, resulting from disease and trauma [1]; the gold standard choice to replace missing teeth is represented by dental implants, allowing normal function, contour, comfort, and speech, restoring the oral health near to normal limits [2]. The success of an implant can be assessed by means of the bone resorption quantification: not more than 1.5 mm in the first year after the placement, not more than 0.2 mm a year in the following period [3]; failures in the integration of the implant material with the natural bone (i.e., osseointegration) are often associated with poor bone quality and/or quantity, which provokes poor anchorage and stability of the implant itself [4].

Currently, implant positioning is more and more frequent (because of more frequent dental caries, the increasing incidence of tooth loss, and the rising aging population); the numbers increased more than tenfold from 1983 to 2002, and fivefold from 2000 to 2005, while they keep growing [5]. According to the European Federation of Periodontology, it is expected that there will be an increase in implant-related diseases up to 2025, despite the improvement in surgical techniques [6]. Consequently, high amounts of money are involved (i.e., some billion dollars [7,8]).

In fact, in spite of all the developments in dental implantology techniques, peri-implant diseases are frequent, resulting from an imbalance between bacterial load and host defense [9]. The identification of the disease is made by observing the deviations from the healthy features of peri-implant tissues, including their dimensions and composition [10]. These pathologies can be classified into two main categories [11]:

1. Peri-implant mucositis [12], an inflammatory lesion of the mucosa surrounding the dental implant;
2. Peri-implantitis [13], including not only gingivitis, but also the loss of supporting bone around the implant [14,15], often associated with suppuration and deepened pockets [9].

Data about prevalence on implant-treated subjects are rare [9], but from some reviews (2008 and 2013), it results that mucositis occurs in about 63–80% of the subjects and in 31–50% of the implants, while peri-implantitis occurs in 19–56% of the subjects and in 10–43% of implant sites [16]. More recent studies present different prevalence data: 19.83% of the subjects and 9.25% of the implants for peri-implantitis, 29.48% of the subjects and 46.83% of the implants for mucositis (2017) [17]; 17% of the subjects and 11% of the implants for peri-implantitis (2018) [18]. However, important numbers are involved and there is an urgency for an effective treatment strategy.

The main characteristics of peri-implantitis are bone loss, inflammation of soft tissue (i.e., gingiva and connective tissue in general) and bacterial infection (which determines bacteria adhesion to the implant surface and abutment, with consequent immune reaction) [11,19]. It can lead to the complete loss of osseointegration and is still the main cause of implant failure, since no completely effective therapies are acknowledged so far [20,21]. There is a general agreement that the treatment of peri-implant disease must include anti-infective measures [9], since it is associated with biofilms [22] of oral microorganisms, which are difficult to eliminate by means of mechanical treatments used alone [23]. Thus, over the years, other techniques were proposed, like antibiotics and antiseptics, but their effect is limited, reducing bleeding on probing and probing depths but not curing the disease [23]. Laser therapy shows minor beneficial effects, but further evaluations on this approach are needed [24]; this is a similar case for ultrasound, which seems to have potential in tissue repair [25] but is not yet widely applied for treatment (whereas opportunities for diagnostic ultrasound in dentistry are growing, as well as in implantology [26]). Therefore, it seems that the outcome of non-surgical treatments is unpredictable [9]. On the other hand, surgical treatment of peri-implantitis consists in open debridement (i.e., removal of infected tissue) and decontamination, in order to cure the inflammatory lesion. This technique was experimented on animals and humans, but the available evidence is extremely limited [27] and the success rate is not satisfactory. Not even regenerative procedures are able to fix the problem; in fact, they limit themselves to fill the osseous defect [9] and there is no evidence of additional beneficial effects.

Hence, the success rate of current peri-implantitis therapies is not satisfying, such that, at present, prevention is the only means to contrast peri-implantitis [19].

Due to the unsatisfactory outcomes of the aforementioned therapies and the absence of a widely accepted treatment [28], an alternative therapy would be desirable. It seems that electricity and electromagnetic (EM) radiation could have good potential in the peri-implantitis field. Indeed, electromagnetic therapy was demonstrated to have different effects on biological tissues:

1. Anti-inflammatory effect, associated with the content of lipid messengers in phospholipids of immunocompetent cell membranes [29]. It produces a decrease in the exudative edema and hyperthermia, comparable to that obtained by means of the administration of therapeutic doses of anti-inflammatory drugs [30];
2. Antibacterial effect (there is the evidence that high-frequency and low-intensity electromagnetic (EM) irradiation presents antibacterial effects on *Escherichia coli* and other bacteria [31]);
3. Promotion of bone formation and reduction of bone resorption; the underlying mechanisms are not clear, but it is likely that pulsed EM irradiation increases DNA synthesis, alters the cellular calcium content in osteoblasts, and can also improve the differentiation of mesenchymal stem cells, which enhance the synthesis of extracellular matrix and the mineralization in osteoblast-like cells [19].

Moreover, EM irradiation also reinforces the effect of some antibiotics and anti-inflammatory drugs by changing metabolic pathways and membranes [19].

It is worthy to underline the fact that the EM signal acts just on the principal hallmarks of peri-implantitis: soft tissue inflammation, peri-implant bone loss, and bacterial growth. Hence, EM signal can be evaluated as a possible therapy for peri-implantitis disease; in the literature, there is a clinical study (started in 2002) exploring this possibility [32] and the same hypothesis was reported in Reference [19]. Therefore, it appears that the development of a novel therapeutic device based on EM radiation would be very promising for peri-implantitis treatment and would also have a great impact on the electromedical device market.

On the other hand, at present, the diagnosis of peri-implantitis disease is commonly made by observing the color and the swelling of the gingiva, the bleeding, the probing depth of peri-implant pockets, or suppuration, as well as sometimes by means of X-rays (to measure the bone height around the implant) [33]. It is easy to understand that the subjectivity and the experience of the clinician play pivotal roles in the outcome of the diagnostic procedure, furnishing results hardly comparable due to their variability. However, in the literature, there is evidence of a relationship between bioimpedance and tissue characteristics, including their electrical properties and their physiological/pathological status; the interest in bioimpedance measurement is growing, proven by the increasing literature on this topic [34,35]. In particular, the composition of the tissue can be characterized if the measurement is performed in a very localized and precise way [36], allowing valuable applications of body impedance analysis (BIA) [37,38]. Brendle et al. [39] used bioimpedance to detect tissues boundary as a control during the bone cement removal in total hip replacement operation. Indeed, the bioimpedance method can be useful to diagnose an inflamed area of tissue when compared with the corresponding healthy one, employed as a control [40].

The authors previously deployed numerical simulations to investigate the possibility of applying the bioimpedance method in dentistry, by comparing healthy and inflamed tissues [41,42]; the obtained results allowed defining the main constraints for the design of a diagnostic system based on electrical bioimpedance measurement. Moreover, preliminary measurements on three patients were carried out, reporting significant differences in impedance modulus (34% and 55% in case of simple inflammation and peri-implantitis, respectively [43]). Furthermore, the realized numerical model helped to link the anti-inflammatory effect to the electric current passing through the soft tissues, whereas the bone healing is accelerated by the electric field, as well documented in the literature [44,45].

For all the reasons reported above, a research project aimed to design an electromedical device took place between 2013 and 2016, ending in the realization of a first prototype of the device, including both a diagnostic and a therapeutic part [46]. The efficacy of the electromagnetic treatment provided was already proven by the authors, reporting a success rate of 81% (on a total of 81 dental implants—30 molars, 23 premolars, nine canines, and 10 incisors) [32]. The clinical evidence of the treatment, together with results from numerical simulations, as well as an accurate consideration of electromagnetic compatibility (EMC) susceptibility aspects, guided the whole design process.

The aim of this paper is to present a novel electro-medical device for the treatment of peri-implant diseases (mucositis and peri-implantitis), which is currently successfully used for therapy, as well as both the numerical simulations and the EMC pre-compliance tests that the authors performed to obtain useful indications for the optimal design of the device. This paper is organized as follows: Section 2 presents the device, reporting its block diagram and the final device; Section 3 describes EMC pre-compliance tests to optimize the design from a susceptibility point of view; Section 4 deals with numerical simulations of the therapy; Section 5 presents the clinical data related to the use of the device; finally, Section 6 presents the authors’ conclusions.

## 2. Materials and Methods

**Figure 1:**
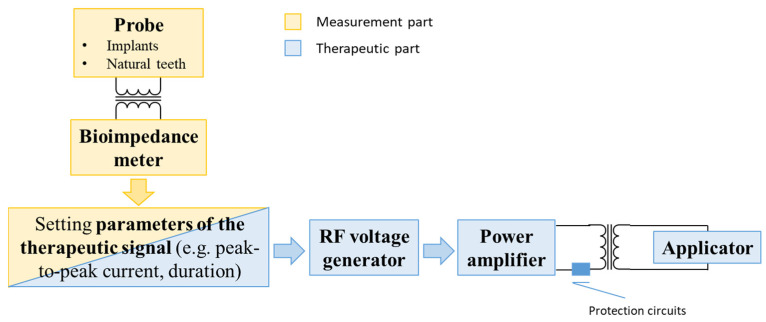
Block diagram of the device.

The electromedical device was designed with the intended use to treat peri-implant pathologies, based on the provision of a proper electromagnetic signal. This is a Class II device, with applied parts of type BF (i.e., floating), working with radio frequency (RF). Three main technical standards were taken into consideration: IEC 60601-1, IEC 60601-2-2, and IEC 60601-1-2. A block scheme of the new device is reported in Figure 1.

The apparatus consists of two different parts, having distinct scopes:

1. Therapeutic aim: the treatment of peri-implant tissues affected by peri-implantitis is performed by means of a radio frequency (RF) electric current (312.5 kHz);
2. Diagnostic aim: the localization and quantification of inflamed tissues are made by means of bioimpedance measurement.

Proper insulation transformers are used both in the measurement and in the therapeutic parts, in order to guarantee the electric safety of the patient.

Both the therapy delivery and the measurement performing are carried out using two electrodes: the active electrode and return (or neutral or passive) electrode. Two different types of probes are implemented, in order to have the flexibility of adapting to dental implants/natural tooth use cases. In the former case, the active electrode is screwed to the implant (in order to establish a good electrical connection), whereas the return electrode is kept in contact with the gingiva; in the latter case, both the electrodes are put in contact with the gingiva.

As mentioned in the previous section, the bioimpedance measurement permits to focus the radio frequency alternating current in the impaired area, since the current densities and pathways from an electrical signal are determined to a considerable extent by the electric properties of biological tissues [39]. Depending on the measured bioimpedance values and, thus, on the severity of the pathology, the therapeutic signal can be properly set to tune the treatment to the pathological conditions of the peri-implant tissues. The therapy consists of the application of a radio frequency (i.e., 312.5 kHz) alternating current burst, with a standard time duration of 120 ms and a peak power value of 6 W, passing through the tissues between the electrodes [32].

With regard to safety aspects, the device includes different MOPs (i.e., “protection circuits”) controlled by a microprocessor, in order to protect the patient and the operator against possible electric hazards.

**Figure 2:**
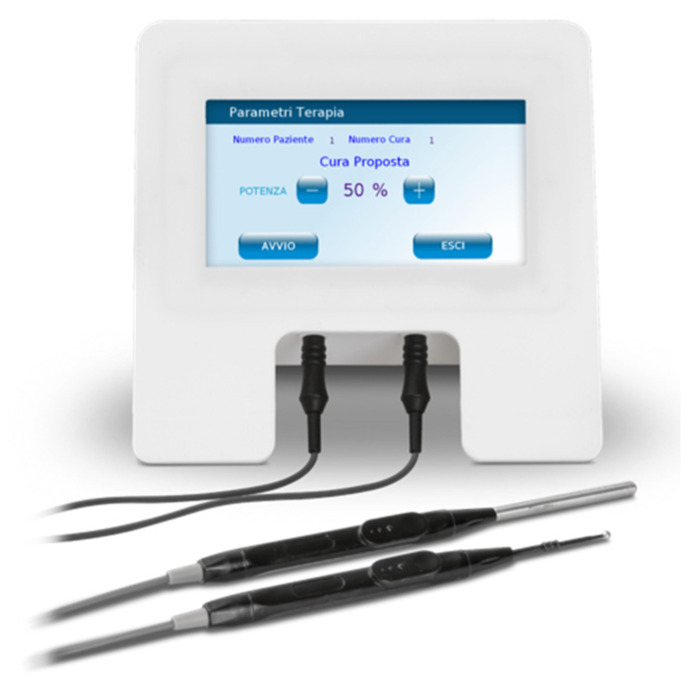
Picture of the market-ready device.

The realization of the electromedical device was translated in a compact, certified, and market-ready final version [47] (Figure 2). It is possible to observe the touch screen, representing the user interface for setting measurement/therapeutic parameters, in addition to the connections for the two electrodes (active electrode—thinner, to focus the therapeutic signal on the area to be treated; electrode of return—with a wider surface, to obtain a lower electric current density).

The treatment consists of the delivery of a RF electric current, whose amplitude can be adjusted dependent on the disease severity (which is quantified through bioimpedance). In particular, the amplitude of the therapeutic signal can be varied between a minimum of 20% and a maximum of 90% of the device nominal value (with a pitch of 5%), limiting the maximum peak current (with dedicated resistors in the circuitry, in order to avoid too high intensities on the tissues); the burst duration is equal to 120 ms.

The device also permits the provision of analgesia before the real treatment, in order to avoid possible pain to the patient. The amplitude of these signals is adjustable in the current version of the device; however, in the future, the choice will be automated after the device calibration on the basis of the clinical conditions of a test population wide enough. The excitation signal used for bioimpedance measurement is at 5 kHz and the result provided is the average value among multiple measures (performed in the set time interval). If anomalous values are obtained (out of physiological ranges, for example, because of non-good electrical contact between electrodes and tissue), an alert is given, and the measurement should be repeated.

The device allows storing the data in each therapeutic session, in order to have information related to each patient’s clinical history.

## 3. EMC Pre-Compliance Tests

In order to be able to sell a medical device in the European Union, it is necessary to comply with the applicable directives and to obtain a European Conformity (CE) marking, certifying that the instrument meets the legal requirements of the European Community. Prior to the marketing of the medical device, it is necessary to submit it to specific standard tests certifying its electromagnetic compatibility (i.e., EMC tests). A set of EMC pre-compliance tests were performed at the laboratories of Università Politecnica delle Marche, in order to perform all the necessary bettering actions to improve the design of the device and obtain a robust final system before its test in a certified EMC laboratory. In fact, pre-compliance tests are useful to get early information on the equipment capacity to meet the requirements when full compliance testing is carried out. Whilst not fully conforming to the requirements of the relevant standards, pre-compliance tests should still be carried out in a manner which will give meaningful results [48], even if there is no a specific definition of pre-compliance testing. As regards the electromagnetic susceptibility (EMS), the authors decided to test the radiated immunity to electromagnetic fields, the immunity to electromagnetic discharge (ESD), and the conducted immunity when a fast transient burst (FTB) is injected in the feeding network. As regards the emissions, conduced and radiated measurements were performed, but two considerations must be highlighted. The first one is that the device is connected to the feeding network only in the charging mode, so the conduced emission test can be carried out only in an idle state. The second consideration concerns the radiated emission measurement. Even if the laboratory environment is not a standard test site and, therefore, the measurements cannot give an absolute value of the radiated electromagnetic field, they can nevertheless give important design indications on the behavior of the apparatus.

It is important to highlight that the case of the device is made of plastic, unable to perform an effective shielding action; thus, the proper electrical layout is essential to guarantee correct and safe operation. Pre-compliance tests were used to drive all the design stages.

All these tests proved to be very significant to determine the most susceptible elements of the apparatus, especially the electrostatic discharge (ESD) susceptibility tests. Once the necessary modifications were proposed and implemented in the final version, the apparatus passed at first attempt the complete tests according to European standard EN 60601-1-2, necessary for the CE marking, saving in this way both time and money.

### 3.1. Radiated EMS Test

To check the immunity of the device the electromagnetic fields according to the European Standard EN 50082-1, the open TEM cell of our laboratory was used; it works properly in a single TEM mode up to 200 MHz (the cut-off frequency of the first higher mode is 230 MHz).

The attention was focused on two subsystems: the circuit that performs the measurement of the periodontal tissue impedance and the circuit that generates the waveform of the discharges during the working mode. In the layout design, great care was used to minimize the circuit dimensions to reduce the area of the current paths, which are the most critical access points for external electromagnetic interferences.

**Figure 3:**
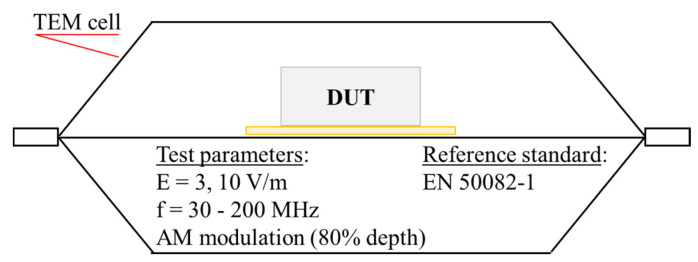
Device under test (DUT) position in the TEM cell.

As depicted in Figure 3, the device was placed into the working volume of the TEM cell, and the entire frequency range from 30 to 200 MHz was examined using 100 frequency steps, generating an electric field of amplitude 3 and 10 V/m and amplitude modulated (AM) by a 1-kHz sine wave (80% modulation depth). According to European Standard 61000-4-3, with the device being non-life-supporting equipment, the electromagnetic field intensity for an immunity-radiated test has to be 3 V/m; the authors decided to expose it also to higher EM intensities (10 V/m) in order to have more information on its eventual weakness to radiated disturbances. As expected, in this frequency range, more than two decades beyond the maximum operating frequency of the device (312.5 kHz), no Electromagnetic interference (EMI) problem was reported.

Since the TEM cell allows us to reduce the frequency of the interferent signal, the device was also tested in a frequency range close to its working frequency (312.5 kHz–3.125 MHz) to stress the electronic circuits, even if not foreseen by EMC regulations. No malfunction was also observed in this case.

### 3.2. Fast Transient Burst Test

The designed apparatus is connected to the feeding line only in the charging mode; therefore, its conduced susceptibility can be tested only in this modality. It is worth remembering that, in charging mode, the device is forbidden from delivering power to the patient. However, this allows us to verify if the device is immune to a hypothetic burst disturbance that could occur in charging mode and that could cause damages to the internal circuitry due to the high intensity of the burst pulses.

**Figure 4:**
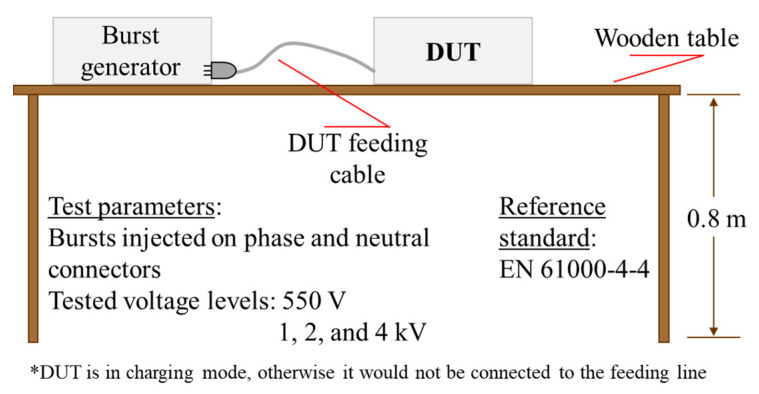
Fast transient burst test.

According to the European standard EN 61000-4-4, the device was placed onto a wooden test table (0.8 m height) and its feeding cable connected to the burst generator Peter Hofbauer Electronic GmbH BNG-900 (Figure 4), placed at a distance of 10 cm above the ground reference plane. Bursts were injected on phase and neutral connectors, since the grounding conductor is not present. All four levels (550 V, 1 kV, 2 kV, and 4 kV) of voltage recommended by the standard were tested. Thanks to the proper choice of the EMI filters and their correct connection, the device exhibited no failure events.

### 3.3. Electrostatic Discharge (ESD) Test

**Figure 5:**
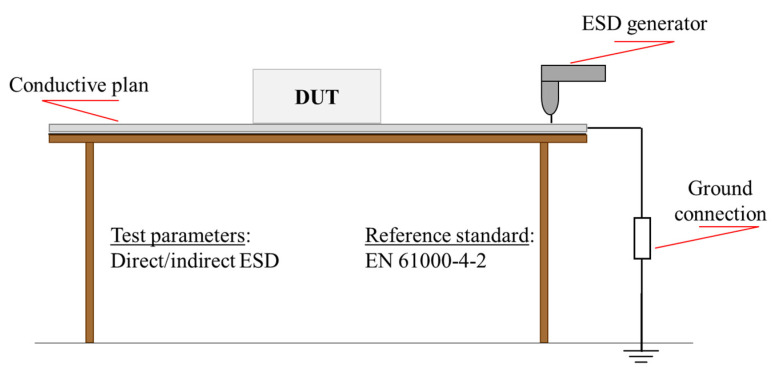
Electrostatic discharge (ESD) test set-up.

An ESD susceptibility test was performed because it is very significant to check the robustness of a device to electromagnetic interferences [49], since it is a broadband phenomenon simultaneously involving both radiated and conduced coupling mechanisms, thus stressing the immunity of the device. According to European standard EN 61000-4-2, both direct and indirect ESD were injected using the ESD generator the Schaffner NSG 432 (with the Schaffner contact discharge adaptor for the direct ESD tests). The device was placed onto a conductive plan, connected to the ground through two 490-kΩ resistors (Figure 5).

The ESD test failed (criterion C) for direct ESD level 3 (6 kV) and for indirect ESD level 3 (8 kV). In fact, the monitor switched off and a hardware reset was necessary to restore the normal behavior of the device. To solve these issues, the authors examined the hardware layout of the device and noticed two weakness points:

The ribbon cable connecting the touch screen to the main board was too long and, therefore, folded. This configuration increased the susceptibility to the electromagnetic field due to the ground current generated by the injection of the ESD in the conductive plane. The ribbon was shortened and two clamps of ferrite were placed at its edges to reduce the induced common voltage noise.

The device had no metallic parts, for safety reasons in a medical environment; however, on the edge between the screen and the dielectric case, a direct ESD happened when the voltage was greater than 6 kV. The authors decided to increase the insulation by inserting a 5-mil (0.127 mm) thick dielectric, which did not affect the performance of the touch screen.

When these two improvements were implemented, the device passed all the ESD tests, on air up to 15 kV and direct up to 8 kV. Having a fully insulated case of the device allows avoiding issues related to ESD.

### 3.4. Radiated Emission Test

**Figure 6:**
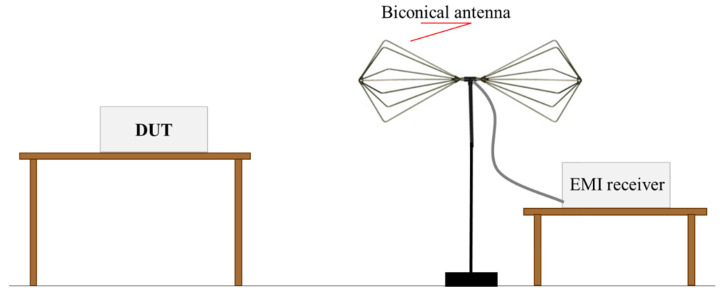
Radiated emission test set-up.

Although the measurement environment was not a standard anechoic test site, the authors decided to also carry out the radiated emission measurement to have an idea of the device behavior in terms of radiated disturbance, to check the presence of electromagnetic noise emitted by the device that could have degraded the performance of other devices placed into the surrounding environment. An EMI receiver (Spectrum Analyzer HP 8568B, RF Preselector HP 85685A and Quasi Peak Adapter HP 85650A) connected to an EMCO Biconical 3104 antenna was used (Figure 6).

When the therapeutic signal was provided, a peak of emission in the frequency range of 35–45 MHz was measured by the receiver. This problem was attributed to the length of the cables carrying the signal from the device to the electrodes, which behaved as an efficient antenna. The test was repeated after twisting the cables connecting the applicators to the body of the device; the radiated emission was reduced by approximately 70% (−10 dB) thanks to the compensation effect attributable to the created twists (adjacent twists radiate field in phase opposition).

## 4. Numerical Simulation of the Therapy

**Figure 7:**
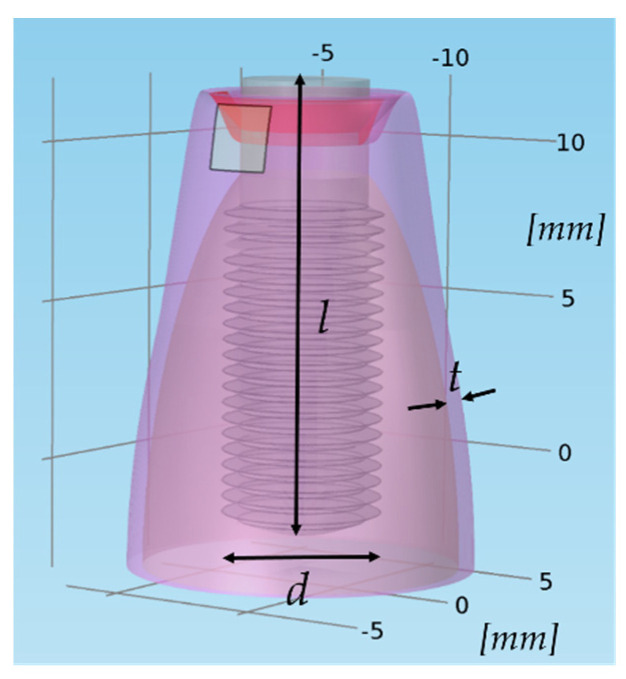
Model geometry of the dental implant screwed in the jawbone: d is the implant diameter (4 mm), l is its length (14 mm), and t is the gingiva thickness (1 mm); the red portion of the gingiva, near the implant, is the inflamed one.

Numerical simulation tools are widely use in bioengineering to optimize the design and customization of biomedical devices [50]. A three-dimensional (3D) model (Figure 7) was realized to numerically simulate the current generated during the normal working condition of the device in COMSOL Multiphysics^®^ environment. A dental implant (realized in titanium) replacing a second premolar tooth root [51] (14 mm in length, 4 mm in diameter) was modeled according to a simplified geometry (a more detailed one could be obtained by means of a reconstruction process based on computer tomography or magnetic resonance tomography volumetric data [52], but the computational load would be very high); it is screwed into the jawbone and the peri-implant tissues are affected by peri-implantitis.

The following tissues were considered: alveolar bone, gingiva (considered more generally as connective tissue), and inflamed gingiva (typical of peri-implantitis); their electrical properties (i.e., electrical conductivity, relative permittivity, and relative permeability) were taken from the literature [53,54,55] and are reported in Table 1; the electrical conductivity of the inflamed gingiva was considered as twice the healthy gingiva one [40], because of hyperemia and infiltration of the adjacent tissues typical of an inflammatory process. For the sake of simplicity, no changes in geometries were considered, even if the inflammatory process is also characterized by edema [56].

The geometry was discretized with a tetrahedral mesh, denser near the edges and around the corners; the model had 340,754 tetrahedral, 63,325 triangular, and 2833 edge elements. The simulation was run with a stationary solver, for a fully coupled solution, in a frequency domain study.

**Figure 8:**
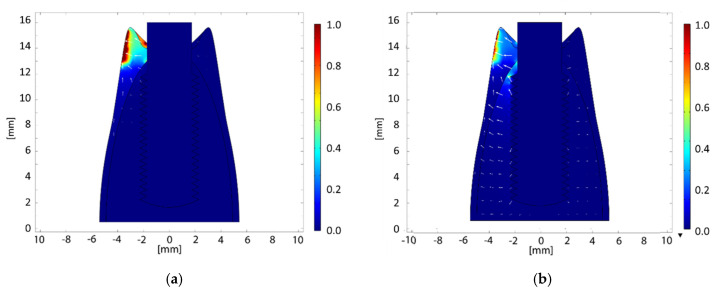
(**a**) Distribution of electric current density (frontal section); (**b**) distribution of electric field (frontal section). Values are normalized with respect to the maximum.

Numerical simulations allowed obtaining the electric current and field distributions resulting from the therapy procedure. The distributions of electric current density and electric fields are reported in Figure 8a,b, respectively; the values are normalized to the maximum, which can vary depending on the selected therapeutic parameters (the maximum electric current amplitude is 200 mA) which is nevertheless limited to the tissues to be treated). The active electrode is the implant itself, while the passive one adheres to the gingiva. It is possible to infer that the anti-inflammatory effect is probably related to the electric current (which crosses the soft tissue, having a higher electrical conductivity), focusing on the inflamed area; on the other hand, bone healing is probably accelerated by the electric field, whose lines pass through the hard tissues, stimulating bone formation [44,45].

### Safety Aspects

**Figure 9:**
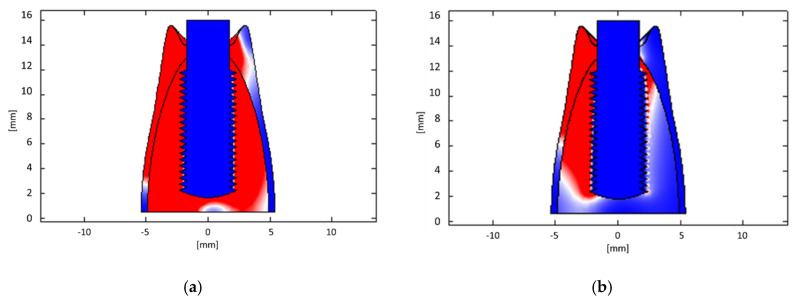
Regions above (red) and below (blue) ICNIRP limits for SAR value (2 W/kg) for (**a**) a maximum intensity therapeutic signal and (**b**) a reduced one (25%).

**Figure 10:**
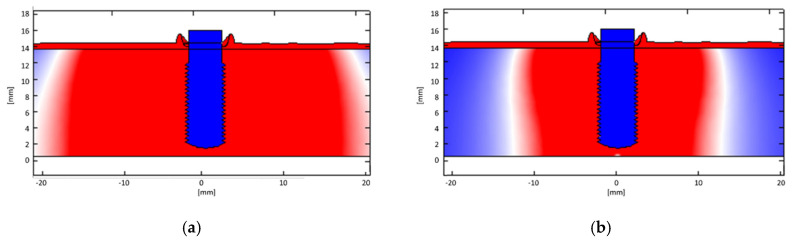
Regions above (red) and below (blue) ICNIRP limits for electric current density value (625 mA/m^2^) for (**a**) a maximum intensity therapeutic signal and (**b**) a reduced one (25%). A dental arch adjacent to the implant is considered.

From the results of numerical simulations, it is possible to observe that the therapeutic signal is substantially confined between the electrodes, without involving the other tissues, consequently demonstrating the safety of the treatment. In fact, in the darkest region of the model map (Figure 8) (i.e., bone), both the electric current density and the electric field values are negligible, leading to electric field and Specific Absorption Rate (SAR) values in the regions not considered by the treatment respecting International Commission on Non-Ionizing Radiation Protection (ICNIRP) [57] guidelines. In Figure 9 and Figure 10, there are distinctions between the areas above (red zone) and below (blue zone) the ICNIRP limits for SAR value (2 W/kg [58]) and electric current density (f/500, with f = 312.5 kHz, i.e., 625 mA/m^2^ [58]), respectively, considering the maximum amplitude for the therapeutic signal and a reduced one (25%). It is possible to notice how the therapeutic signal is confined in the tissue area to be treated, ensuring the safety of the treatment.

**Figure 11:**
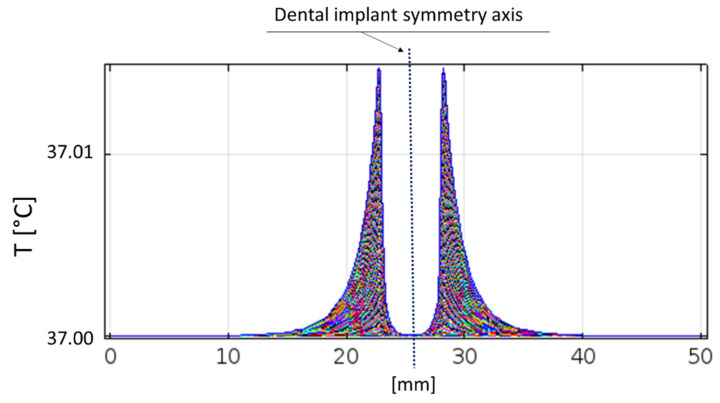
Temperature increase in correspondence with the horizontal line just above the implant thread (i.e., the horizontal line at a height of 12 mm in Figure 8).

Moreover, it is worthy to consider that, at frequencies higher than 100 kHz, the heating effect is the mainly relevant one, since neither nervous nor muscular stimulations are reported in the literature and no ventricular fibrillations occur [59]. It is possible to note that the temperature increase is irrelevant just above the implant thread, even if the adjacent dental arch is considered (Figure 11); in fact, tissues remains at 37 °C.

Therefore, it is possible to state that the therapy provided by the developed device does not involve the tissues not considered by the treatment, neither in terms of nervous/muscular stimulation nor in terms of thermal effects, proving to be safe and in compliance with ICNIRP guidelines.

## 5. Demonstration of the Clinical Efficacy of the Apparatus

**Figure 12:**
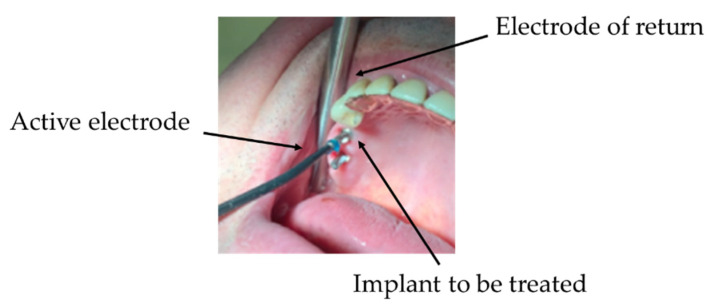
The electromagnetic treatment on a patient; the active electrode is screwed to the implant, whereas the neutral one is put in contact with the adjacent gingiva.

As observable in Figure 12, the two electrodes should be correctly positioned in correspondence with the implant to be treated; the active electrode is screwed to the implant itself, whereas the return one is kept in contact with the gingiva.

In the case of mucositis, after a standard treatment of oral hygiene, the electromagnetic therapeutic signal is repeatedly provided 3–4 subsequent times at the maximum power of the device; the patient is re-evaluated after two weeks and the treatment is repeated, if necessary. On the other hand, in the case of peri-implantitis, a standard treatment of oral hygiene and antibiotics is administered to the patient before the treatment, which should be repeated every 3–4 days for two weeks; then, a two-week break is observed. The patient is re-evaluated after two weeks; if there are improvements, the treatment is repeated (one-month cycles); when the evaluation parameters (i.e., pain, swelling, bleeding, exudate presence, implant mobility, radio-transparency) are definitely boosted, the treatment is stopped and the patient is re-evaluated every three months; when no enhancements are observed, surgical treatment would be required (supported by further electromagnetic treatment, where appropriate). If no signs of improvement are evident, the implant should be removed.

The clinical efficacy of the treatment provided by the developed device (commercialized in June 2019, with the commercial name PeriCare^®^) was demonstrated during the first year of application on a treated population of approximately 200 patients. The success rate reported was nearly 100% in the case of mucositis and >80% for peri-implantitis (with the exception of long-duration peri-implantitis, where the success rate was >50%). As success criteria, different evaluation parameters were considered: swelling, pain, bleeding, presence of exudate, implant mobility, radio-transparency; it is worthy to note that probing depth was not considered, in accordance with the latest recommendations on classifications for peri-implant disease and conditions [60]. All the treated implants reported a reduction in bleeding on probing, plaque index, and probing depth, indicating complete success of the mucositis treatment.

Positive effects on treated patients can be observed from the reported pictures and radiographic images, showing the peri-implant tissues before and after the treatment, together with the evaluation of the parameters reported above. Four clinical cases are reported hereafter as examples; it is worthy to note that they come from a wider clinical study (started when the new developed device was commercialized), whose results will be published in a dedicated study when the dataset is completed.

**Figure 13:**
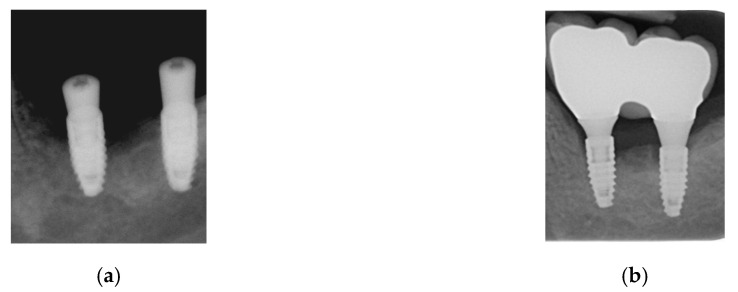
Clinical case No. 1: dental implant (**a**) before and (**b**) after the treatment.

The first reported clinical case is a 48-year-old man reporting swelling and pain 15 days after the implant positioning (before the loading phase). A follow-up X-ray was used to plan the treatment with the newly developed device. After four therapeutic sessions (one week apart from each other, up to 90% of the maximum power), the symptoms disappeared and the implant was fully recovered, as also visible from the follow-up X-ray (Figure 13). Two months later, after a further follow-up X-ray, the prosthetic finalization was made (loading phase).

**Figure 14:**
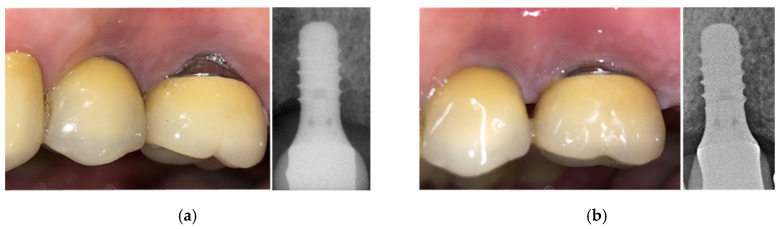
Clinical case No. 2: dental implant (**a**) before and (**b**) after the treatment.

The second clinical case is a 69-year-old man with pain and bleeding on probing. Following a session of oral hygiene, the patient was treated with the new developed device. After four therapeutic sessions (two per week, each consisting of seven consecutive therapeutic signal deliveries, up to 90% of the maximum power), the implant could be considered recovered and pain disappeared after the first therapeutic session (Figure 14). Monthly check-ups were planned and follow-up X-rays confirmed the positive outcome of the therapy.

**Figure 15:**
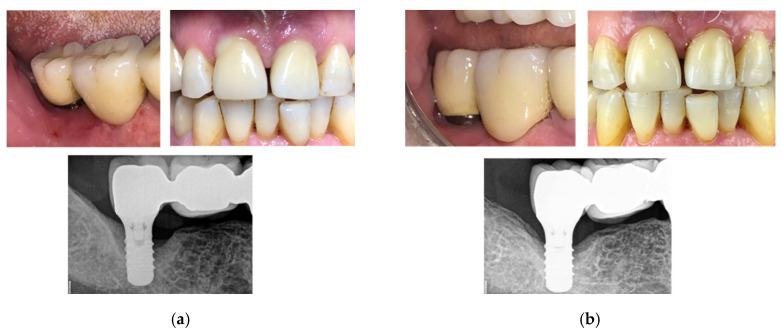
Clinical case No. 3: dental implant (**a**) before and (**b**) after the treatment.

The third clinical case is a 75-year-old man with swelling, bleeding on probing, and exudate presence. Following a session of oral hygiene, the patient was treated with the new developed device. After four therapeutic sessions (two per week, with increasing intensities—up to 70%, 80%, 90%, and 90% of the maximum power in the first, second, third, and fourth sessions, respectively; four consecutive signal deliveries were used in the first three sessions, with seven in the last one), swelling was reduced and bleeding on probing was resolved. Continuing the treatment with monthly cycles, the implants were recovered after approximately eight months (Figure 15). Periodical check-ups were planned.

**Figure 16:**
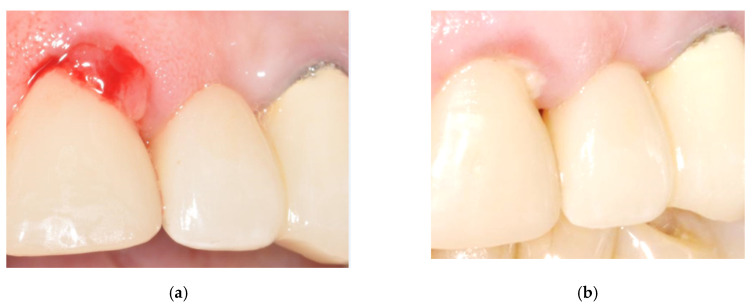
Clinical case No. 4: dental implant (**a**) before and (**b**) after the treatment.

The fourth clinical case is a 75-year-old woman with bleeding and exudate presence. After two therapeutic sessions (one week, at 90% of the maximum power), tissues conditions definitely improved and the exudate presence was clearly reduced after two weeks (Figure 16).

In all the cases, the patient-reported outcomes were evaluated, underling that the first effect of the treatment was recognized in the immediate disappearance of pain and symptoms perceived by the patient.

It is important to underline that a correct management of peri-implant mucositis can prevent the development of peri-implantitis disease [61], thus enhancing the longevity if the implant itself. In the literature, non-surgical treatments of mucositis are said to be effective without the additional use of chemical/mechanical agents, even if the complete resolution of inflammatory conditions seems not to be reached [62].

## 6. Discussion and Conclusions

In this paper, the authors presented an electromedical device designed with both therapeutic and diagnostic aims, especially in the application fields of peri-implantitis and mucositis. In particular, the developed device is capable of measuring the tissue electrical bioimpedance (related to their health status) and providing a proper RF electric current that has beneficial impacts on impaired tissues, namely, an anti-inflammatory and anti-bacterial effect, promotion of bone formation, and reduction of bone resorption. Thanks to the capability of measuring the tissue bioimpedance, the therapeutic signal can be adjusted according on the basis of the actual conditions; on the other hand, bioimpedance can locate the area where the treatment should be focused, thus avoiding the involvement of healthy tissues. Furthermore, bioimpedance measurements allow monitoring the disease discourse and consequently adapting the therapeutic strategy. Furthermore, in the literature, bioimpedance measurements were applied with similar aims. Tornuev et al. [40] considered the measurement of bioimpedance to evaluate tissues, since inflammatory processes are characterized by hyperemia, extravasation of plasma, and infiltration of the adjacent tissues, determining a significant presence of liquids, which increase the electric conductivity of the tissue. Meanwhile, the quantification of edema and changes in blood flow by means of bioimpedance were also evaluated in [63] for the application of a wearable sleeve to put around the knee joint. Therefore, the measurement of bioimpedance can help the clinician in the diagnostic process, quantifying the tissue health status (especially relating to inflammations), as well as allowing him to monitor the course of the pathology after one or more therapeutic sessions. A diagnostic instrument based on the measurement of bioimpedance allows the clinician to objectively diagnose the impaired tissue (also in terms of inflammation severity), thereby focusing the therapy on the inflamed area, thus limiting the involvement of surrounding tissues. Such an approach would also be applicable in different fields, including all treatments targeted to inflammatory diseases (e.g., magnetotherapy or hyperthermia).

Numerical simulations provided valuable indications for the device design (both on the diagnostic and the therapeutic parts), as well as EMC pre-compliance tests. In fact, since for the commercialization of a new electromedical device, specific EMC standards have to be respected, some preliminary measurements were carried out on the device at the laboratories of Università Politecnica delle Marche during the design phase, in order to take EMC susceptibility aspects into account following the early development of the system. Pre-compliance tests have the important task of highlighting EMC problems due to weaknesses in the electromagnetic layout occurring during the design and realization stages, before submitting the device to a notified body. With some caution, expert people can carry out measurements, understand results, and suggest improvements even if the test site does not show the characteristics required by the standards. In particular, the authors would like to outline that, according to their experience, the most significant and severe test is the ESD one, because the discharge intensity, its wide spectral content, and the choice of the injection points are able to stress most of the weakness points of the examined device, resulting in a consistent saving of time and money. In fact, thanks to the accurate design and realization processes, the device was able to comply with all the required standards and is on the market since June 2019, currently being used in approximately 50 dental offices, all reporting decidedly satisfactory success rates (approximately 98% and 80% for mucositis and peri-implantitis, respectively, evaluated according to clinical objective observations of swelling, pain, blooding, probing, and X-ray examination, together with bioimpedance measurement). The obtained success rates are even more relevant if compared with the state of the art. Indeed, peri-implant disease resolution is rarely reported; Leonhardt at al. addressed a success rate of surgical treatment (consisting of open debridement and decontamination) equal to 58% with the additional use of antibiotics [64], and regenerative procedures are not able to fill the osseous defects [9]. Regarding mucositis, non-surgical treatment can be useful to solve the symptoms, with the exception of inflammatory conditions, which seem to remain unresolved even after therapy [62]. On the contrary, the newly developed device acts on the inflammatory characteristics and proves to be almost completely effective (98% success rate).

It is worthy to underline that the developed electromedical device can be useful not only in peri-implantitis therapy, but also in the case of inflammations occurring around a natural tooth which should be treated (i.e., periodontitis [65]), since the inflammation mechanism is the same.

In the future, all the data gathered in clinical practice should be used together with artificial intelligence (AI) technologies to mark a tissue as inflamed or not, independently of the comparison with a corresponding healthy tissue. This is particularly important when measurement on healthy tissue is not possible, since comparing two dental implants is not always feasible in clinical practice. With a large amount of training data, the quantification of disease severity could also be possible. To this aim, it would be of utmost importance to systematically build a database with clinical data from patients treated in different centers, as well as different countries, in order to be able to make distinctions linked to different ethnicities, in addition to different types of dental implants. Measurement results on healthy tissues should also be included, to expand the database and improve the AI algorithm’s predictive power. Independently of AI applications, such a database would also be a valuable tool to improve the statistical significance of recorded data for the automatic setting of therapeutic parameters on the basis of the measured data.

Finally, the application fields of the apparatus can potentially be extended. Different body parts affected by inflammation-related pathologies could be considered; on the other hand, the use of such a device in dentistry could be extended to the monitoring of bone level in dental implants, since a correctly osseointegrated implant provides a higher bioimpedance value, due to growth at the tissue–implant interface [66,67]. Moreover, the stability of the implant could be evaluated considering the phase of the measured impedance [68].

## Figures and Tables

**Table 1:** Biological tissue electric properties at 312.5 kHz (i.e., therapeutic signal frequency).

Tissue	σ (S/m)	ε_r_	μ_r_
Enamel	0.021	190	1
Dentine	0.022	190	1
Pulp	0.390	347	1
Gingiva	0.390	245	1
Inflamed gingiva	0.780	245	1
Bone	0.021	190	1
